# A Capacitive Feedback Transimpedance Amplifier with a DC Feedback Loop Using a Transistor for High DC Dynamic Range

**DOI:** 10.3390/s20174716

**Published:** 2020-08-21

**Authors:** Jung-hoon Noh

**Affiliations:** Agency for Defense Development, P.O. Box 35, Yuseong, Daejeon 34134, Korea; jhnoh@korea.ac.kr

**Keywords:** capatitive feedback, transimpedance, high dynamic DC, system stability, transistor

## Abstract

This study proposes a capacitive feedback transimpedance amplifier (CF-TIA) using a transistor in the direct current (DC) feedback loop for high DC dynamic range. In some applications, the background DC input can vary widely from the minimum to the maximum, and TIA have to sense the target signal even on the top of the maximum DC input. In a conventional CF-TIA, however, the allowable DC input is constrained by the value of the resistor in the DC feedback loop. To allow a fairly high DC input, the resistor is set to a very low value. This causes the thermal noise current to increase significantly. The increased thermal noise is always present even in the minimum DC input, thus degrading the overall noise performance. The circuit proposed herein overcomes this shortcoming by using the transistor instead of the resistor. The adverse effect of the parasitic capacitance of the transistor on system stability is compensated for as well. Then, the analyses of the overall frequency response and design parameters, including the cut-off frequency and attenuation ratio associated with system stability, are presented for the proposed circuit. In addition, in order to cope with the problem that stability is dependent on the amount of DC input, a simple method for ensuring system stability regardless of DC component value is introduced. The presented analyses and the method are generalized for all CF-TIA applications.

## 1. Intoroduction

Precision instrumentation systems, such as optical receivers [1], electrical sensors [2,3,4,5], emerging biosensors [6,7,8] photodetectors [9,10,11], and other current-output measurement systems, often contain a transimpedance amplifier (TIA). An operational amplifier (op-amp) with negative feedback is typically used in a TIA. The most typical TIA topology is a resistive feedback TIA (RF-TIA), which is simple and easy to analyze as the feedback resistor directly matches the transimpedance gain. In [3,4,6,7,12,13,14,15], a capacitive feedback TIA (CF-TIA) has been proposed to reduce the thermal noise generated by the feedback resistor, as well as overcome difficulties in the integration of high resistance in complementary metal-oxide-semiconductor (CMOS) chips.

**Figure 1:**
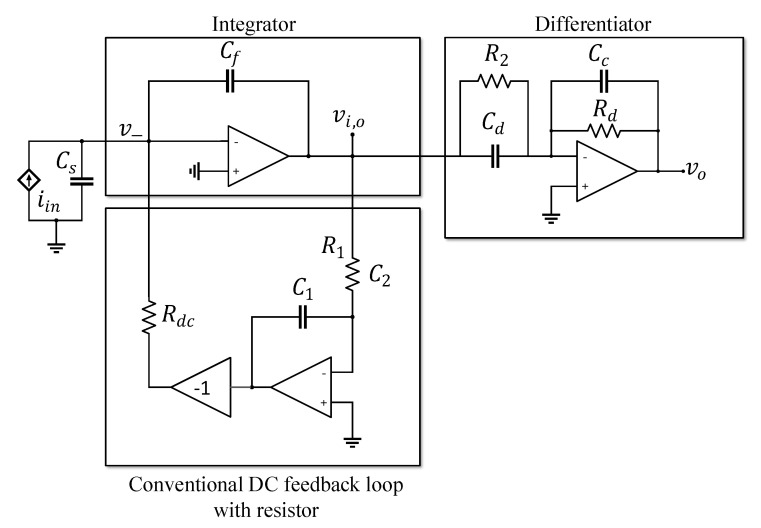
Capacitive Feedback Transimpedance Amplifier with conventional DC feedback loop.

The basic topology of the CF-TIA is composed of an integrator and a cascaded differentiator, as shown in Figure 1. The DC feedback loop is typically inserted in the first stage integrator. It provides a DC path to prevent saturation of the integrator’s feedback capacitor $C_{f}$ and simultaneously to bias the op-amp. The conventional DC feedback loop consists of an non-inverting integrator to filter the DC components on the feedback path and a resistor $R_{dc}$ to drain the DC components from the input node. Although a high value of $R_{dc}$ is preferred to reduce the thermal noise currents of $R_{dc}$, it limits the maximum allowable DC input with the output voltage range of the op-amp, as in [6,7]. The voltage drop across $R_{dc}$ with the DC input should be less than the op-amp output voltage range which is usually slightly less than the op-amp supply voltage. The higher the allowable DC input, the lower the value of $R_{dc}$.

In some applications such as optical sensors, the DC input varies according to the amount of background light. While the normal DC input from the ambient light is typically low, the maximum feasible value of the DC input can be fairly high when intense light is directly incident on the sensors. The TIA has to sense the weak target signal on top of the expected maximum value of the DC input in the worst case scenario. However, to allow for high DC input, the value of $R_{dc}$ should be very low, causing the thermal noise to increase significantly. To make matters worse, increased thermal noise is always present even at normal or low DC inputs, degrading overall system performance. In this study, to overcome the shortcomings of the conventional CF-TIA, a new topology that replaces $R_{dc}$ with the transistor in the feedback loop is introduced. The method of discharging DC inputs using the transistor in the DC feedback loop is one of the widely used methods in various circuits, but it has not yet been used and analyzed for CF-TIA. With the transistor, the high DC can flow with a much smaller voltage drop across the base-emitter (gate-drain) compared to the significant voltage drop that occurs when a resistor is used. The thermal noise of the resistor is then replaced by the shot noise of the transistor.

Actually, when assuming a constant DC input, the shot noise is larger than the thermal noise. However, if the DC dynamic range, i.e., the range from the minimum to maximum DC, is significant, the proposed topology has the benefits in the overall noise. The shot noise of the proposed topology varies with the amount of the DC input. In normal cases, DC inputs are much less than the maximum value, so the proposed topology can exhibit lower noise than conventional topology that always shows the worst thermal noise to cope with maximum DC inputs. The advantage in terms of the noise is more distinct compared to the CMOS implementation of the conventional CF-TIA where the thermal noise and shot noise coexist for the pseudo-resistor [6,7]. For the CMOS implementation, the proposed topology shows the lower noise than the conventional topology by the amount of thermal noise even for the maximum DC input.

The proposed circuit includes a method for compensating for the adverse effect of the parasitic capacitance of the transistor on system stability. The overall frequency response and design parameters, such as the cut-off frequency and attenuation ratio associated with the system stability, are presented and analyzed for the proposed topology. Moreover, the inclusion of an additional capacitor to the DC feedback loop for ensuring system stability regardless of the DC input value is discussed. Through simulations and experiments, the proposed CF-TIA scheme is validated. In this study, the circuit is implemented with discrete components, but the frequency response model and stability analysis presented are generalized to be applicable to all CF-TIA applications and CMOS chip designs.

## 2. CF-TIA with DC Feedback Path Using Transistor

**Figure 2:**
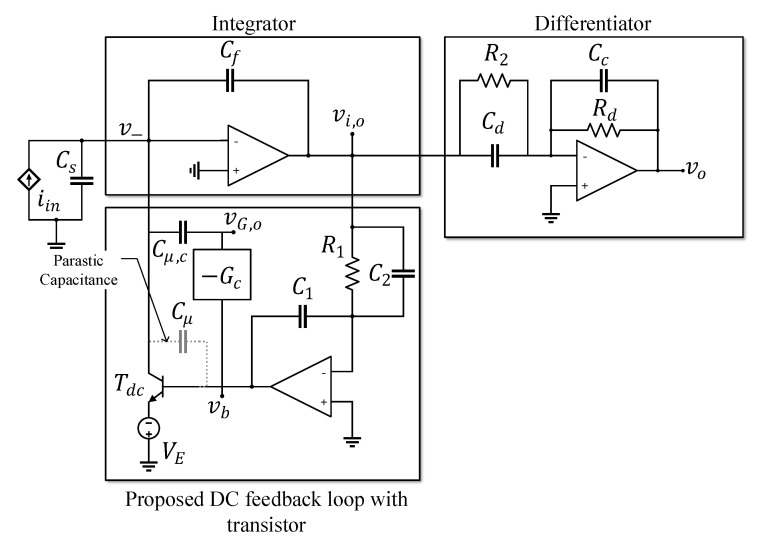
Capacitive Feedback Transimpedance Amplifier with the proposed DC feedback loop with a transistor.

This section investigates and analyzes the proposed CF-TIA using a transistor in the DC feedback loop shown in Figure 2. The transistor $T_{dc}$ serves as a variable current sink that pulls the average DC input $I_{dc}$ from the signal path under a steady state condition. Note that the high current can flow from the collector (drain) to the emitter (gate) with only a low base-emitter (gate-drain) voltage.

First, the fundamental performance of the CF-TIA is presented. The achievable bandwidth of the CF-TIA or the upper cutoff frequency, $f_{H}$, is limited by the gain-bandwidth product of the op-amp $f_{GBWP}$ and the ratio between $C_{f}$ and $C_{in}$ as follows [6,7]:

(1) $$f_{H}\leq f_{GBWP}\cdot \frac{C_{f}}{C_{in}+C_{f}},$$

where $C_{in}=C_{s}+C_{i,op}+C_{\mu }+C_{\mu ,c}$ is the total capacitance at the TIA input including the sensor capacitance $C_{s}$, the input capacitance of the op-amp $C_{i,op}$ (encapsulating the differential and common mode capacitance), the base-collector (gate-drain) parasitic capacitance of the transistor $C_{\mu }$, and the capacitor $C_{\mu ,c}$ to compensate the effect of $C_{\mu }$.

Following this, the overall flat gain of the generic CF-TIA can be described as follows:

(2) $$\frac{v_{o}}{i_{in}}=\frac{C_{d}R_{d}}{C_{f}},$$

where $C_{d}$ and $R_{d}$ constitute the second differentiator, $i_{in}$ is the input current, and $v_{o}$ is the output voltage of the CF-TIA. Because the gain of the differentiator increases with the frequency until it is rolled off by the open-loop gain of the op-amp, the product of $C_{d}$ and $R_{d}$ is constrained as follows:

(3) $$C_{d}R_{d}\leq \frac{f_{GBWP}}{2\pi f_{H}^{2}}.$$

Note that while both a bipolar junction transistor (BJT) and a field-effect transistor (FET) can be used as $T_{dc}$, the FET shows a higher parasitic capacitance $C_{\mu }$ than that of the BJT, resulting in a reduced bandwidth as in (1). Thus, we use the BJT for $T_{dc}$ here. Then, in order for $T_{dc}$ to be in an active mode, the appropriate emitter voltage $V_{E}$ must be set such that the collector-emitter voltage is greater than 0.7 V. Moreover, to compensate for the influence of $C_{\mu }$ on system stability, the inverting amplifierG(s) whose overall gain is $-G_{c}$, and the capacitor $C_{\mu ,c}$ are inserted between the collector and the base of $T_{dc}$.

A detailed analysis of the frequency response of the proposed CF-TIA is presented in next. Applying Kirchhoff’s current law at the negative input node of the integrator gives [16]:

(4) $$\begin{array}{ccc} i_{in} & = & sC_{f}\left(v_{-}-v_{i,o}\right)+sC_{s}v_{-}+\left(v_{-}-\frac{v_{i,o}}{sC_{1}R_{1}}\right)sC_{\mu }+\left(v_{-}-G_{c}\frac{v_{i,o}}{sC_{1}R_{1}}\right)sC_{\mu ,c}\\ & \overset{\left(a\right)}{=} & -v_{i,o}\left(\frac{s\left(C_{f}+C_{s}+C_{\mu }+C_{\mu ,c}\right)}{A(s)}+sC_{f}+\frac{G_{c}C_{\mu ,c}-C_{\mu }}{C_{1}R_{1}}+\frac{g_{m}}{sC_{1}R_{1}}\right)\\ & \overset{\left(b\right)}{\approx } & -v_{i,o}\left(sC_{f}+\frac{\beta }{C_{1}R_{1}}+\frac{g_{m}}{sC_{1}R_{1}}\right) \end{array}$$

where $v_{i,o}$ is the output voltage of the first stage integrator, A(s) is the open-loop gain of the op-amp, $g_{m}$ $\left(=I_{dc}/V_{T}\right)$ is the transconductance of $T_{dc}$, $I_{dc}$ is the DC input, $V_{T}$ is the thermal voltage (approximately 25 mV at a room temperature of 259 K), $C_{1}$ and $R_{1}$ constitutes the integrator in the DC feedback loop, and $\beta =G_{c}C_{\mu ,c}-C_{\mu }$. In (4), (a) follows from substituting $v_{-}$ as $-v_{i,o}/A\left(s\right)$, and the approximation (b) follows from that $\left|A(s)\right|$ is exceedingly high within the system bandwidth.

By rewriting (4) to the transimpedance gain form, the transfer function of the integrator $H_{i}\left(s\right)$ is obtained as follows:

(5) $$H_{i}\left(s\right)=\frac{v_{i,o}}{i_{in}}\approx -\frac{\frac{1}{C_{f}}s}{s^{2}+\frac{\beta s}{C_{1}R_{1}C_{f}}+\frac{g_{m}}{C_{1}R_{1}C_{f}}},$$

Rewriting (5) to a standard form of the transfer function of a second-order bandpass filter with a center frequency $w_{0}$ and a damping ratio ζ(=1/(2Q)) yields:

(6) $$H_{i}\left(s\right)=-\frac{C_{1}R_{1}}{\beta }\frac{2\zeta w_{0}s}{s^{2}+2\zeta w_{0}s+w_{0}^{2}}$$

where $w_{0}=2\pi f_{0}$,

(7) $$f_{0}=\frac{1}{2\pi }\sqrt{\frac{g_{m}}{C_{1}R_{1}C_{f}}}\;\mathrm{and}\;\zeta =\frac{\beta }{2\sqrt{g_{m}C_{1}R_{1}C_{f}}}.$$

Now, we obtain the upper and lower cut-off frequencies, $f_{i,H}$ and $f_{i,L}$ of $H_{i}\left(s\right)$. From the fact that $f_{0}$ is the geometric mean of $f_{i,H}$ and $f_{i,L}$, and from (5), followings are derived:

(8) $$f_{i,H}\cdot f_{i,L}={\left(\frac{1}{2\pi }\right)}^{2}\frac{g_{m}}{C_{1}R_{1}C_{f}}\;\mathrm{and}\;f_{i,H}+f_{i,L}=\frac{\beta }{2\pi C_{1}R_{1}C_{f}}.$$

When the wide passband is assumed as $f_{i,H}\gg f_{i,L}$, $f_{i,H}+f_{i,L}\approx f_{i,H}$. Thus, $f_{i,H}$ and $f_{i,L}$ can be expressed as follows:

(9) $$f_{i,H}=\frac{\beta }{2\pi C_{1}R_{1}C_{f}}\;\mathrm{and}\;f_{i,L}=\frac{g_{m}}{2\pi \beta }.$$

Moreover, from the assumption of wide passband by $\zeta \gg 1/\sqrt{2}$, $f_{i,H}\gg f_{i,L}$ is proved, expressed as

(10) $$\frac{\beta }{2\pi C_{1}R_{1}C_{f}}\gg \frac{g_{m}}{\pi \beta }>\frac{g_{m}}{2\pi \beta }.$$

From the expressions for β and $H_{i}\left(s\right)$ in (5), it can be observed that both $G_{c}$ and $C_{\mu ,c}$ ensure the circuit stability. In the absence of $G_{c}$ and $C_{\mu ,c}$, β becomes negative, resulting in two positive real poles in $H_{i}\left(s\right)$. If the system has any poles with a positive real part, the part of outputs diverges without a bound, causing system instability. The value of $C_{\mu ,c}$ is preferred to be negligibly small relative to the total input capacitance in order to maximize the achievable bandwidth as in (1).

In terms of stability, $G_{c}$ is preferred to be high, so that makes the system free of gain peaking as $\zeta \geq 1/\sqrt{2}$, even with the high $g_{m}$. Note that we assume that the stability is determined based on a condition of a maximally flat response (Butterworth response), which is $\zeta =1/\sqrt{2}$. However, $G_{c}$ is limited by the condition that the first pole frequency of G(s) should be placed above $f_{0}$. Note that $f_{0}$ varies with $I_{dc}$, and the case when $f_{0}$ exceeds the system bandwidth $f_{H}$ is not taken into account. The aforementioned discussion suggests the following conditions:

(11) $$C_{\mu ,c}\ll C_{in}\;\mathrm{and}\;G_{c}\leq \frac{f_{GBWP}}{f_{H}}.$$

**Figure 3:**
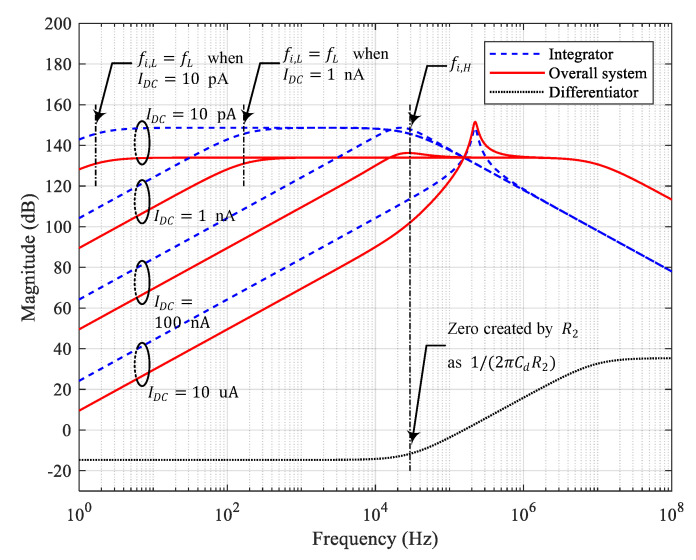
Transfer functions of the integrator, differentiator, and the overall system without $C_{2}$ for $I_{dc}=10$ pA, 100 nA, 1 nA, and 10 uA, where $C_{\mu }=1$ pF, $C_{\mu ,c}=1$ pF, $G_{c}=50$, $C_{f}=0.2$ pF, $C_{d}=1$ nF, $C_{c}=8.51$ pF, $R_{d}=2$ kΩ, $R_{2}=8.16$ kΩ, $C_{1}=10$ nF, and $R_{1}=100$ kΩ.

By cascading the differentiator to the integrator, the overall CF-TIA transfer function, H(s), is derived by multiplying $H_{i}\left(s\right)$ by the differentiator transfer function as follows:

(12) $$H_{d}\left(s\right)=\frac{R_{d}}{R_{2}}\left(\frac{1+sC_{d}R_{2}}{1+sC_{c}R_{d}}\right),$$

where $C_{c}$ is used to stabilize the differentiator as $C_{c}=1/\left(2\pi R_{d}f_{H}\right)$, and $R_{2}$ is placed parallel to $C_{d}$ in order to generate a zero in $H_{d}\left(s\right)$ at $f_{i,H}$ in (9) such that $C_{d}R_{2}=C_{1}R_{1}C_{f}$. Then, the flat gain of $H_{i}\left(s\right)$ is multiplied by $R_{d}/R_{2}$, and the decrease in $H_{i}\left(s\right)$ beyond $f_{i,H}$ is compensated by the increase in $H_{d}\left(s\right)$ with the introduced zero. The resulting H(s) becomes the bandpass filter transfer function, whose lower cutoff frequency $f_{L}$ is equal to $f_{i,L}$. $\left|H_{i}\left(s\right)\right|,\;\left|H_{d}\left(s\right)\right|,$ and $\left|H(s)\right|$ are shown in Figure 3 for increasing $I_{dc}$ from 10 pA to 10 uA. Note that as $I_{dc}$ increases, ζ decreases. Eventually, a gain peaking occurs, as shown for $I_{dc}=10$ uA in Figure 3.

We can include the additional capacitor $C_{2}$ parallel to $R_{1}$ to ensure stability, regardless of the $I_{dc}$ value. In the presence of $C_{2}$, following the approaches in (4) and (5) gives $H_{i}\left(s\right)$ as

(13) $$H_{i}\left(s\right)\approx -\frac{\frac{1}{\left(1+\gamma \right)C_{f}}s}{s^{2}+\frac{1}{1+\gamma }\left(\frac{\beta }{C_{1}C_{f}R_{1}}+\gamma \frac{g_{m}}{\beta }\right)s+\frac{g_{m}}{\left(1+\gamma \right)C_{1}C_{f}R1}},$$

where γ is the parameter that controls the value of $C_{2}$ and system stability, such that $\beta C_{2}=\gamma C_{1}C_{f}$. The design parameters are then described as follows:

(14) $$\begin{array}{ccc} f_{0} & = & \frac{1}{2\pi }\sqrt{\frac{g_{m}}{\left(1+\gamma \right)C_{1}R_{1}C_{f}}}\;\mathrm{and} \end{array}$$

(15) $$\begin{array}{ccc} \zeta & = & \frac{1}{2\sqrt{1+\gamma }}\left(\frac{\beta }{\sqrt{g_{m}C_{1}R_{1}C_{f}}}+\gamma \frac{\sqrt{g_{m}C_{1}R_{1}C_{f}}}{\beta }\right). \end{array}$$

Then, the upper and lower frequencies of $H_{i}\left(s\right)$ are expressed in two cases depending on the amount of $I_{dc}$. The first case is for a low $I_{dc}$ with $g_{m}\ll \beta^{2}/\left(\gamma C_{1}C_{f}R_{1}\right)$, and

(16) $$f_{i,H}=\frac{\beta }{2\pi \left(1+\gamma \right)C_{1}R_{1}C_{f}}\;\mathrm{and}\;f_{i,L}=\frac{g_{m}}{2\pi \beta }.$$

The second case is for a high $I_{dc}$ with $g_{m}\gg \beta^{2}/\left(\gamma C_{1}C_{f}R_{1}\right)$, and

(17) $$f_{i,H}=\frac{\gamma g_{m}}{2\pi (1+\gamma )\beta }\;\mathrm{and}\;f_{i,L}=\frac{\beta }{2\pi \gamma C_{1}R_{1}C_{f}}.$$

Notably, $R_{2}$ is placed to generate a zero in $H_{d}\left(s\right)$ at $f_{i,H}$ of (16) as previously discussed.

Eventually, multiplying $H_{d}\left(s\right)$ of (12) to $H_{i}\left(s\right)$ of (13) renders H(s) to the bandpass filter frequency response whose lower cutoff frequency is expressed as follows:

(18) $$f_{L}=\left\{\begin{array}{c} \frac{g_{m}}{2\pi \beta },\;\;g_{m}\ll \beta^{2}/\left(\gamma C_{1}C_{f}R_{1}\right)\\ \frac{\gamma g_{m}}{2\pi \left(1+\gamma \right)\beta },\;\;g_{m}\gg \beta^{2}/\left(\gamma C_{1}C_{f}R_{1}\right) \end{array}\right..$$

**Figure 4:**
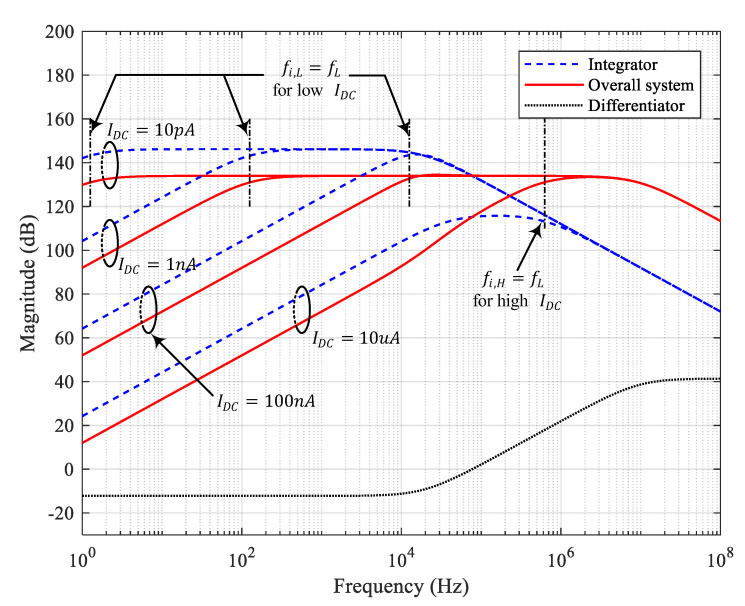
Transfer functions of the integrator, differentiator, and overall system placing $C_{2}=40.8$ pF such that γ=1 for $I_{dc}=10$ pA, 100 nA, 1 nA, and 10 uA, where $C_{\mu }=1$ pF, $C_{\mu ,c}=1$ pF, $G_{c}=50$, $C_{f}=0.2$ pF, $C_{d}=1$ nF, $C_{c}=8.51$ pF, $R_{d}=2$ kΩ, $R_{2}=8.16$ kΩ, $C_{1}=10$ nF, and $R_{1}=100$ kΩ.

Note that the inclusion of $C_{2}$ reduces the overall flat gain magnitude by a factor of 1+γ. To achieve the same flat gain magnitude that is exhibited when $C_{2}$ is not included, either $R_{d}$ or $C_{d}$ should be multiplied by 1+γ. From the arithmetic-geometric mean inequality, ζ of (14) has a lower bound of the following:

(19) $$\zeta \geq \sqrt{\frac{\gamma }{1+\gamma }},$$

where equality holds when $g_{m}=\beta^{2}/\left(\gamma C_{1}C_{f}R_{1}\right)$. For γ=1, the stability is always ensured by $\zeta \geq 1/\sqrt{2}$, regardless of the $I_{dc}$ value, as in Figure 4.

Noise performance analysis is presented in the remaining part of this section. The input-referred noise model is commonly used in noise analysis for comparing input signals and noise levels. In this topology, instead of the thermal noise of the resistor, the current noise of the transistor, $i_{TR}$, is added to the typical root mean square (RMS) value of the input-referred noise expression of [17] as follows:

(20) $$i_{N.rms}=\sqrt{i_{n}^{2}+i_{TR}^{2}+\frac{{\left(e_{n}2\pi f_{c}\left(C_{s}+\beta \right)\right)}^{2}}{3}}.$$

where $i_{n}$ is the inverting-input current noise of the op-amp, and $e_{n}$ is the differential voltage noise of the op-amp. The noise $i_{TR}$ is the shot noise of $T_{dc}$, where $i_{TR}^{2}\approx 2qI_{dc}$ and *q*(=1.6e−19) is the electron charge. Note that a flicker noise can be significant when implementing circuits with CMOS technology or using MOSFET instead of BJT in $T_{dc}$. However, the flicker noise can be made negligible when assuming the broad range of signal bandwidth and using a non-minimal MOSFET area [4].

The proposed CF-TIA shows better overall noise performance compared to that of the conventional CF-TIA when the dynamic range of $I_{dc}$ is wide. For example, assume that the dynamic range of $I_{dc}$ is 30 dB from 100 nA (normal $I_{dc}$) to 100 uA (the expected maxima value of $I_{dc}$). Because of the maximum $I_{dc}$, $R_{dc}$ of the conventional CF-TIA is constrained to 50 kΩ $\left(=V_{s}/100\;\mathrm{uA}\right)$ where the supply voltage $V_{s}$ is 5 V. The maximum output voltage of the op-amp is assumed to be same as the supply voltage. Then, the thermal noise current of $R_{dc}$ becomes 1 pA/$\sqrt{\mathrm{Hz}}$$\left(=\sqrt{4kT/R_{dc}}\right)$ where *k* (=1.38e−23J/K) is the Boltzmann constant and *T* (=300 K) is the absolute temperature.

This thermal noise current always exists even in normal $I_{dc}$ degrading the overall noise performance. However, in the proposed CF-TIA, thermal noise current of the resistor is eliminated and the shot noise of a normal $I_{dc}$ (=100 nA) becomes 0.17 pA$\sqrt{\mathrm{Hz}}$ $\left(=\sqrt{2qI_{dc}}\right)$, which is much lower than the thermal noise current of 1 pA/$\sqrt{\mathrm{Hz}}$.

Furthermore, the noise of the proposed circuit is always less than that of the conventional CF-TIA implemented with CMOS, as in [6,7], in which the thermal noise and shot noise currents coexist. If the conventional CF-TIA is implemented with CMOS technology, the feedback resistor is implemented as a pseudo resistor with MOS devices. Therefore, the input-referred noise expression of conventional CMOS CF-TIA includes not only the thermal noise of feedback resistor, but also the shot noise of the MOS [7]. However, (20) has only the shot noise term without the thermal noise term.

Note that here β is associated with the noise contribution of the input capacitance term, the third term in (20). Both the influence on the total noise and the overall system stability should be considered together when determining $G_{c}$.

## 3. SIMULATION and EXPERIMENT

**Figure 5:**
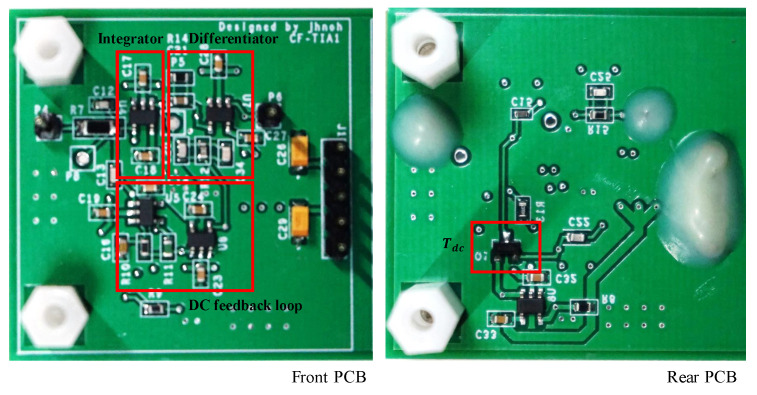
Hardware Implementation.

The presented circuits were implemented and simulated using PSpice to verify the presented analyses of the transfer functions and design parameters. A photograph of the realized circuit is presented in Figure 5. All the circuits were built using the same op-amp (OPA657, Texas Instruments). OPA657 has wideband and low-noise characteristics, and its $f_{GBWP}$ is 1.6 GHz, $A_{ol}$ is 75 dB at room temperature, and $C_{i,op}$ of 5.2 pF. It is to be noted that $f_{GBWP}$ is not a trimmed parameter and can vary by the maximum ±40% due to the process variation for any op-amp [18]. Consequently, even though the datasheet specifies the $f_{GBWP}$ to be 1.6 GHz, I have considered $f_{GBWP}$ to be 1.28 GHz, about 80% of this typical value in order to account for process variations.

**Figure 6:**
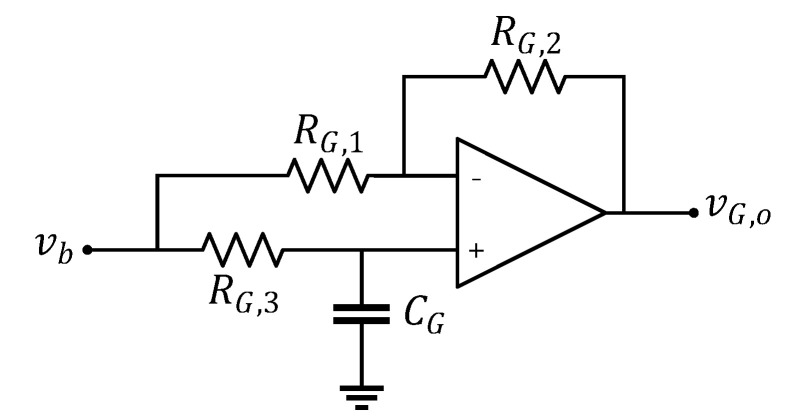
Implementation of G(s). The values of the discrete components are $R_{G,1}=1\;\Omega$, $R_{G,2}=50\;\Omega$, $R_{G,3}=1\;M\Omega$, and $C_{G}=1$ uF.

Note that to compare the simulation, experiment, and analytic results, all values of the discrete components were set equivalently as the assumed values in the analytical example shown in Figure 4. Several variables were set by considering the laser position sensor application and its practical implementation. For the laser position sensor QP154-Q (First Sensor), $C_{s}$ is assumed to be 20 pF. Taking the lowest value of the practical discrete capacitor, $C_{f}$ is set to 0.2 pF. By using MMBT5179 NPN transistor (On Semiconductor) with low parasitic capacitance, $C_{\mu }$ is set to 1 pF. The $C_{d}$ value has an upper limit of approximately 1 nF because of the capacitive loading effect at the OPA657 output. In addition, Figure 6 shows an example of the implementation of G(s) for the simulation and experiment. $R_{G,3}$ and $C_{G}$ render the amplifier AC coupled to prevent $v_{G,o}$ from being saturated with DC components. Given the aforementioned assumptions, the maximum achievable bandwidth was calculated as 9.3 MHz using (1), and overall flat gain magnitude was determined to be 5E+6 from (2).

**Figure 7:**
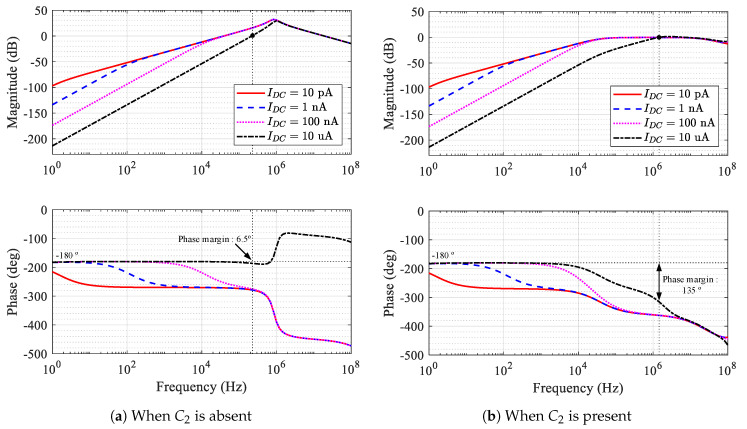
Simulation of integrator’s loop gain.

To assess the stability, the magnitude and phase of the loop gain were obtained through simulation, and plotted in Figure 7. Figure 7 shows the magnitude and phase of the loop gain of the integrator, depending on the presence or absence of $C_{2}$. For low $I_{dc}$, although the phase at low frequencies is about −180°, the stability is ensured by a high gain margin. At high frequencies, the phase drops from −180°, resulting in a very high phase margin at the gain crossover point where the magnitude of the loop gain reaches unity-gain (0 dB). However, as $I_{dc}$ increases, the point at which the phase begins to drop increases significantly. Eventually, for $I_{dc}=$10 uA in Figure 7a, the phase margin is meager at 6.5°, causing the system to become unstable. This result is consistent with the analysis result shown in Figure 3, where the gain peaking occurs with $I_{dc}=10$ uA. In the presence of $C_{2}$, the zero is added before the gain crossover frequency. Thus, we can ensure enough phase margin with $C_{2}$. For $I_{dc}=$10 uA in Figure 7b, the phase margin becomes 135°. This result is also consistent with the analysis result shown in Figure 4, where the gain peaking is prevented on the presence of $C_{2}$ with $I_{dc}=10$ uA.

**Figure 8:**
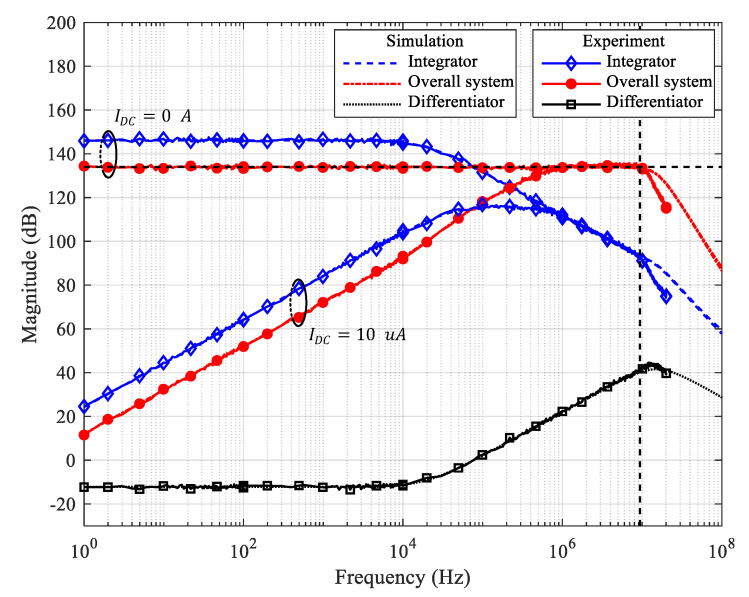
Simulation and Experimental result.

The frequency responses of the simulation and results of the experiment are shown in Figure 8. The experimental results strongly agree with the simulation results as well as the analytical results of Figure 4. The only difference is that the experimental results start to roll off at about $f_{H}$, slightly lower than the roll-off point of simulation results. There can be several reasons why this roll-off point in the measurement results is lower than that in the simulation results, such as additional input capacitance in the PCB layout and variations in the parameters of the circuit components. The major reason appears that the $f_{GPWP}$ of the op-amp used in the actual implementation is less than the ideal values recorded in the datasheet or the simulation model parameters due to the process variation [16].

## 4. Conclusions

In this study, a CF-TIA with a transistor in the DC feedback loop is proposed and analyzed for high DC input dynamic range. Our system avoids the shortcoming of the conventional CF-TIA, whereby the thermal noise for the maximum DC input always present even in normal or low DC input cases. In the proposed circuit, the thermal noise is replaced by the shot noise varying with DC input. Thus, the proposed circuit can have the benefit in overall noise performance for normal or low DC inputs. Moreover, the proposed circuit compensates for the adverse effect of the parasitic capacitance of the transistor, which would otherwise lead the system to diverge with a positive real pole in the frequency response. The overall frequency response and design parameters, such as the cut-off frequency and attenuation ratio associated with system stability, are analyzed providing useful guidelines for the proposed CF-TIA design. Furthermore, a method that avoids gain peaking, regardless of the DC input, is introduced. The proposed CF-TIA and its analyses are validated by the excellent agreement between the analyses, simulation, and experimental results. The proposed circuit and its analyses are not limited to a specific application. They can be applied to various sensor measurements, such as emerging bio-sensors, nuclear science instrumentation, and optical receivers, and their CMOS chip design implementations.
